# Supplementary material for: Exploring the research needs, barriers and facilitators to the collection of biological data in adolescence for mental health research: a scoping review protocol paper
Source: BMJ Open. 2024 Jun 11;14(6):e081360. doi: 10.1136/bmjopen-2023-081360 (PMC11168127; doi:10.1136/bmjopen-2023-081360)
Supplement: Supplementary data [file bmjopen-2023-081360supp002.pdf]

SUPPLEMENTARY MATERIAL:
Plain language summary

This plain language summary has been co-written with young people from our Youth Expert Working Group

Adolescence happens between the ages of 10 and 19 when moving from childhood to adulthood. Adolescence is a time when a person goes through many changes both physically and mentally and is the time when a person may be more vulnerable to having mental health problems, such as depression and/or anxiety. There are lots of things that can increase the chances of mental health problems happening, like genetics, stressful things happening during childhood, or coming from a disadvantaged background. But we still don't know much about how biology plays a role in all of this.

Scientists have done some biological research into mental health during adolescent years, but it's quite limited for different reasons such as the groups who fund research being concerned that young people will not want to take part. Because of this, we don't fully understand how biology mixes with other things like stress, environment, and social factors in adolescence, and to do this we need more research. Therefore, it's important for us to understand what is stopping us from doing this kind of research, and how we can make it easier for young people, like you, to get involved.

We are doing a 'scoping review', which is looking at all the studies published previously, to help us to find out more about this issue. A scoping review looks at how much research has been done on the topic and whether there are any gaps in our understanding. This paper explains how we will conduct this review (the so-called "protocol").

We will be searching different databases to find studies that focus on mental health, and that have looked at people between the ages of 11 and 18. We are especially interested in studies that have collected biological information like blood samples or brain scans.

We will look at all the studies that are relevant and see how they relate to the question we are trying to answer. We will be looking at things like how hard it is to get young people to participate in biological studies, how scientists work with communities, and why some groups of people might be more or less likely to take part in this type of research than others. We will also look for gaps in our knowledge, where none of the previous studies have answered certain questions on this topic.

All of the research we find will be drawn together and summarised to help us understand what we know so far about the difficulties of doing research with young people looking at biology and mental health.
