# Supplementary material for: Exploring the research needs, barriers and facilitators to the collection of biological data in adolescence for mental health research: a scoping review protocol paper
Source: BMJ Open. 2024 Jun 11;14(6):e081360. doi: 10.1136/bmjopen-2023-081360 (PMC11168127; doi:10.1136/bmjopen-2023-081360)
Supplement: Supplementary data [file bmjopen-2023-081360supp001.pdf]

APPENDICES:

Appendix I: Search strategy for PubMed database

1. ("MRI" OR "fMRI" OR "EEG" OR "PET" OR "neuroimaging" OR "biological research" OR "biological sample*" OR "biologic*" OR "blood sample* " OR "urine sample*")

AND

2. ("mental health" OR "mental" OR "depression" OR "anxiety" OR "self harm" OR "self-harm" OR "suicide" OR "psychiatric" OR "psychopathology" OR "mood disorder" OR "autism" OR "neurodivergence")

AND

3. ("youth*" OR "adolescent" OR "adolescence" OR "teen*" OR "teenager*" OR "high school student*" OR "AYA")

AND

4. ("retention" OR "recruitment" OR "recruit" OR "recruited" OR "enroll" OR "enrol" OR "enrollment" OR "enrolment" OR "enrolling" OR "enrolled" OR "participation")

Appendix II: Data extraction form

CELEBRATE – WP1 Scoping review data extraction template (version 1)

Date form completed (dd/mm/yyyy)	
Reviewer name	
Citation	

Authors	
Year of publication	
Country of origin	
Indication (eg/ depression or anxiety)	
Aims	
Methodology/study design	
Sample size	
Sample age	
Sample socio-demographics	
Biological measures (list)	
Biological outcomes (list)	
Recruitment details	
Retention details (no. of withdrawals)	
Young people engagement strategies described	
Stakeholder engagement described	
Community engagement described	
Feedback strategies described	

Notes:	
