# Supplementary material for: Exploring the research needs, barriers and facilitators to the collection of biological data in adolescence for mental health research: a scoping review protocol paper
Source: BMJ Open. 2024 Jun 11;14(6):e081360. doi: 10.1136/bmjopen-2023-081360 (PMC11168127; doi:10.1136/bmjopen-2023-081360)
Supplement: Author's manuscript [file bmjopen-2023-081360.draft_revisions.pdf]

BMJ Open

BMJ Open is committed to open peer review. As part of this commitment we make the peer review history of every article we publish publicly available.

When an article is published we post the peer reviewers' comments and the authors' responses online. We also post the versions of the paper that were used during peer review. These are the versions that the peer review comments apply to.

The versions of the paper that follow are the versions that were submitted during the peer review process. They are not the versions of record or the final published versions. They should not be cited or distributed as the published version of this manuscript.

BMJ Open is an open access journal and the full, final, typeset and author-corrected version of record of the manuscript is available on our site with no access controls, subscription charges or pay-per-view fees (http://bmjopen.bmj.com).

If you have any questions on BMJ Open's open peer review process please email info.bmjopen@bmj.com

BMJ Open

Exploring the research needs, barriers, and facilitators to the collection of biological data in adolescence for mental health research: A Scoping Review Protocol paper

Journal:	*BMJ Open*
Manuscript ID	bmjopen-2023-081360
Article Type:	Protocol
Date Submitted by the Author:	25-Oct-2023
Complete List of Authors:	Worrell, Courtney ; King's College London, Department of Psychological Medicine, Institute of Psychiatry, Psychology, and Neuroscience<br>Pollard, Rebecca; King's College London, Department of Psychological Medicine, Institute of Psychiatry, Psychology, and Neuroscience<br>Weetman, Tyler; University of Birmingham, Institute for Mental Health<br>Sadiq, Zara; University of Birmingham, Institute for Mental Health<br>Pieptan, Maria; King's College London, Department of Psychological Medicine, Institute of Psychiatry, Psychology, and Neuroscience<br>Brooks, Gillian; King's College London, King's Business School<br>Broome, Matthew ; University of Birmingham, Institute for Mental Health ; Birmingham Women's and Children's NHS Foundation Trust<br>Campbell, Niyah; University of Birmingham, Institute for Mental Health<br>Gardner, Nzinga; National Institute for Health and Care Research, Public Review Member<br>Harding, Seeromanie; King's College London, Department of Population Health Sciences, Faculty of Life Sciences and Medicine<br>Lavis, Anna; University of Birmingham, Institute of Applied Health Research<br>McEachan, Rosemary; Bradford Institute for Health Research<br>Mondelli, Valeria; King's College London, Department of Psychological Medicine, Institute of Psychiatry, Psychology, and Neuroscience<br>Morgan, Craig; King's College London, Health Service and Population Research Department, Institute of Psychiatry, Psychology, and Neuroscience; King's College London, ESRC Centre for Society and Mental Health, Institute of Psychiatry, Psychology, and Neuroscience<br>Nosarti, Chiara; King's College London, Department of Child and Adolescent Psychiatry, Institute of Psychiatry, Psychology and Neuroscience; King's College London, Centre for the Developing Brain, Department of Perinatal Imaging and Health, School of Biomedical Engineering and Imaging Sciences<br>Porat, Talya; King's College London, Department of Population Health Sciences, Faculty of Life Sciences and Medicine; Imperial College London, Dyson School of Design Engineering, Faculty of Engineering<br>Ryan, David; Bradford Institute of Health Research<br>Schmid, Lea; King's College London, Department of Psychological Medicine, Institute of Psychiatry, Psychology, and Neuroscience<br>Shire, Katy; Bradford Institute of Health Research<br>Woods, Anthony; King's College London, Department of Psychological Medicine, Institute of Psychiatry, Psychology, and Neuroscience

Pariante, Carmine; King’s College London, Department of Psychological Medicine, Institute of Psychiatry, Psychology, and Neuroscience
CELEBRATE Youth Expert Working Group , CELEBRATE Youth Expert Working Group ; CELEBRATE project
Dazzan, Paola; King’s College London, Department of Psychological Medicine, Institute of Psychiatry, Psychology, and Neuroscience
Upthegrove, Rachel; University of Birmingham, Institute for Mental Health; Birmingham Women’s and Children’s NHS Foundation Trust

Keywords: Adolescent, MENTAL HEALTH, Adolescents < Adolescent

SCHOLARONE™
Manuscripts

Exploring the research needs, barriers, and facilitators to the collection of biological data in adolescence for mental health research: A Scoping Review **Protocol paper**

Courtney Worrell[1], Rebecca Pollard[1], Tyler Weetman[2], Zara Sadiq[2], Maria Pieptan[1], Gillian Brooks[3], Matthew R Broome[2,4], Niyah Campbell[2], Nzinga Gardner[5], Seeromanie Harding[6], Anna Lavis[7], Rosemary R C McEachan[8], Valeria Mondelli[1], Craig Morgan[9,10], Chiara Nosarti[11,12], Talya Porat[6,13], David Ryan[8], Lea Schmid[1], Katy Shire[8], Anthony Woods[1], Carmine M. Pariante[1], CELEBRATE Youth Expert Working Group, Paola Dazzan[1], Rachel Upthegrove[2,4]

Affiliations:

1. Department of Psychological Medicine, Institute of Psychiatry, Psychology, and Neuroscience, King's College London, London, UK
2. Institute for Mental Health, University of Birmingham, Birmingham, UK
3. King's Business School, King's College London, London, UK
4. Birmingham Women's and Children's NHS Foundation Trust, Birmingham, UK
5. National Institute for Health and Care Research (NIHR) Public Review Member, UK
6. Department of Population Health Sciences, Faculty of Life Sciences and Medicine, King's College London, London, UK
7. Institute of Applied Health Research, University of Birmingham, Birmingham, UK
8. Bradford Institute of Health Research, Bradford, UK
9. Health Service and Population Research Department, Institute of Psychiatry, Psychology, and Neuroscience, King's College London, London, UK
10. ESRC Centre for Society and Mental Health, Institute of Psychiatry, Psychology, and Neuroscience, King's College London, London, UK
11. Department of Child and Adolescent Psychiatry, Institute of Psychiatry, Psychology and Neuroscience, King's College London, London, UK
12. Centre for the Developing Brain, Department of Perinatal Imaging and Health, School of Biomedical Engineering and Imaging Sciences, King's College London, London, UK
13. Dyson School of Design Engineering, Faculty of Engineering, Imperial College London, London, UK

Author emails:

courtney.worrell@kcl.ac.uk
rebecca.pollard@kcl.ac.uk
tyler.weetman@nhs.net
zara.sadiq4@nhs.net
maria.pieptan@kcl.ac.uk
gillian.brooks@kcl.ac.uk
m.r.broome@bham.ac.uk
n.campbell@bham.ac.uk
nzinga.g@gmail.com
seeromanie.harding@kcl.ac.uk
a.c.lavis@bham.ac.uk
Rosie.McEachan@bthft.nhs.uk
valeria.mondelli@kcl.ac.uk
craig.morgan@kcl.ac.uk

For peer review only - http://bmjopen.bmj.com/site/about/guidelines.xhtml

chiara.nosarti@kcl.ac.uk
t.porat@imperial.ac.uk
David.Ryan@bthft.nhs.uk
lea.schmid@kcl.ac.uk
Katy.Shire@bthft.nhs.uk
tony.woods@kcl.ac.uk
carmine.pariante@kcl.ac.uk
paola.dazzan@kcl.ac.uk
r.upthegrove@bham.ac.uk

Corresponding author details: Courtney Worrell, Desk G.33.74, The Maurice Wohl Clinical Neuroscience Institute, Cutcombe road, King's College London, London, SE5 9RT. Email: courtney.worrell@kcl.ac.uk, Tel: 0207 848 5440.

For peer review only

ABSTRACT

Introduction: While research into adolescent mental health has developed a considerable understanding of environmental and psychosocial risk factors, equivalent biological evidence is lacking and is not representative of economic, social, and ethnic diversity in the adolescent population. It is important to understand the possible barriers and facilitators to conduct this research. This will then allow us to improve our understanding of how biology interacts with environmental and psychosocial risk factors during adolescence. The objective of this scoping review is to identify and understand the needs, barriers, and facilitators related to the collection of biological data in adolescent mental health research. **Methods and analysis:** Reviewers will conduct a systematic search of PubMed, Medline, Scopus, Cochrane, ERIC, EMBASE, ProQuest, EBSCO Global Health electronic databases, relevant publications, and reference lists to identify studies published in the English language at any time. This scoping review will identify published studies exploring mental health/psychopathology outcomes, with biological measures, in participants between the ages of 11-18, and examine the reported methodology used for data collection. Data will be summarised in tabular form with narrative synthesis and will use the methodology of Levac et al., (2010) [1], supplemented by subsequent recommendations from the Joanna Briggs Institute Scoping Review Methodology [2]. **Ethics and dissemination:** Ethical approval is not required for this scoping review. The scoping review will be conducted with input from Patient and Public Involvement (PPI), specifically including young people involved in our study 'CELEBRATE' ('Co-producing a framework of guiding principles for Engaging representative and diverse cohorts of young people in Biological ReseArch in menTal hEalth' - www.celebrateproject.co.uk) Youth Expert Working Group. Dissemination will include publication in peer-reviewed journals, academic presentations, and on the project website.

STRENGTHS AND LIMITATIONS OF THIS STUDY

- This review is being conducted according to methodological frameworks and guidance such as that reported by Levac et al., (2010) [1] and the Joanna Briggs Institute Scoping Review Methodology [2].
- The systematic search will include grey literature which reduces publication bias.
- The search will include 7 different databases (as well as a manual search of the references) to maximise identification of relevant studies.

- The iterative approach to charting data during extraction ensures that all relevant information will be captured.
- The search is limited to work published in the English language which may exclude some relevant publications.

KEYWORDS: Adolescence; Barriers; Biological measures; Facilitators; Mental health.

INTRODUCTION

Adolescence is a distinct period of significant change. Between the ages of 10-19, adolescence sees a string of changes biologically, socially, and psychologically. In this important timeframe, the development that adolescents undergo has an undeniable impact on longer-term trajectories of health and well-being [3]. Due to these substantial changes, this period provides a window of vulnerability, whereby adolescents may be at increased risk of developing poor mental health.

Mental health problems are a leading cause of health-related disability in children and adolescents, with reports that at least 10% globally experience a mental disorder [4]. The UK Office for National Statistics (2017) suggest that 1 in 8 young people between the ages of 5-19 have experienced at least one form of psychopathology in the UK [5]. In a large UK-based cohort, Cybulski et al., (2021) have reported an increased incidence in the number of young people seeking support for mental health disorders between 2003-2018 [6], suggesting an upward trend, one that has seemingly accelerated in the context of COVID-19. Indeed, following the COVID-19 pandemic, concerns about declining adolescent mental health are now being highlighted in the literature, with a meta-analysis indicating a two-fold increase in symptoms of anxiety and depression [7]. It is important to note, however, that approximately half of the studies included in this meta-analysis represent samples from China, and others representing North America and Asia, leading to arguments that they may not represent the global picture, and those from low- and middle-income countries [8]. According to the World Health Organisation (2021), depression, anxiety, and behavioural disorders, such as Attention Deficit Hyperactivity Disorder (ADHD), are the most common forms of mental disorders experienced by adolescents [9]. Regarding mental disorders in adulthood, the available evidence suggests 75% have onset during adolescence [10] showing clear evidence that adolescence is a critical window, and vulnerability during this time can have a substantial impact on longer-term health, specifically mental health. Poor mental health during this period can additionally have an impact on education, future employment, and other longer-term consequences.

Researchers have explored the possible risk factors for mental health disorders in adolescents, focussing on genetic, environmental, and psychosocial risk factors. Some of these factors include exposure to childhood trauma [11,12], bullying [13,14], and difficult family relationships [15], all of which have been shown to play a role in increasing the risk of developing a mental health problem during adolescence. Other factors include environmental and socioeconomic inequalities [16]. A systematic review including data from 23 countries demonstrated that children and adolescents with socioeconomic disadvantage (based on factors such as household income and poverty), were two to three times more likely to develop mental health difficulties, with a somewhat cumulative effect [17].

Existing large adolescent cohorts with mental health data tend to lack biological measures. Existing cohorts typically explore only saliva or small-volume blood samples for genotyping and basic biomarkers [18]. So, whilst this literature begins to demonstrate why adolescents may develop mental health disorders, there is a considerable gap in knowledge from a biological perspective.

For peer review only

Specifically, in our understanding of how biology interacts with environmental and psychosocial risk factors to impact mental health during adolescence [19].

It is known that adolescents may be resistant to engaging with mental health services, particularly for trauma-related disorders, which has led to assumptions regarding their willingness to become involved in research [20]. Where studies are beginning to explore richer biological measures, recruitment to such studies may encounter several barriers. A school-based pilot study, conducted by Warne et al., (2022) collecting salivary cortisol samples found that only 11.3% of parents provided consent for their child to participate, despite the coproduction of the protocol with key stakeholders including parents and children [21], demonstrating some of the possible barriers which can affect this research. When research in child and adolescent populations includes biological data collection, lower recruitment rates may be due to the burden and acceptability of the research; in a study exploring child and adolescent participation in research, Gattuso et al., (2006) found high rates of refusal to participate, which often related to the use of data collection methods, such as blood sampling, which were perceived to be invasive and burdensome [22]. In a feasibility study, young people were asked to report their subjective experience after completing mock clinical interviews, self-report forms, neuroimaging, and blood samples [23]. Most responses indicated that the young people felt safe in all the assessments, and the assessment viewed most favourably was the mock magnetic resonance imaging (MRI) scan, which the authors noted as an important finding given the assumption that in research settings MRI is perceived as burdensome and distressing [23]. The mock interviews and self-report measures, on the other hand, were often reported as stressful and too long [23]. The topic of research being considered 'too sensitive' was another highly reported reason for refusal which may imply further difficulties when the topic of research surrounds mental ill health [22]. Some studies also demonstrate issues with levels of attrition in such research. Mascarell Maričić et al., (2020) discuss this issue in the context of the IMAGEN cohort, a longitudinal study of 2000 14-year-olds recruited and followed up to the age of 22. While discussing how attrition can have an impact on longitudinal multimodal statistical modelling, the authors note that they were able to still have complete data for 1300 of the cohort by age 22 which provided this particular study an opportunity to still explore the developmental trajectories in focus [24]. However, a review by Dimolareva et al., (2018) argued that it is generally difficult to understand more about the nature of recruitment challenges, non-compliance, and attrition in research with adolescents as many relevant papers fail to report such information [25].

Even in the face of good recruitment targets, a lack of representativeness still hinders the generalisability of findings to adolescent populations and potentially those most at risk. For example, Warne et al., (2022) acknowledged a lack of diversity in the sample recruited in the salivary cortisol study [21]. Predominantly, research looking at biological mechanisms of mental disorders has focused on adults, and there is a sparsity of biological research on mental disorders, such as depression, in adolescence [26]. Looking at the acceptability of biological data collection in children, Condon (2016) highlighted that those who drop out of research or do not participate efficiently, are possibly those who are most important to investigate, tending to be those from under-represented groups [27], and other research highlights that higher levels of attrition are seen in those with lower socioeconomic status or mental health difficulties [28]. However, it should be noted that this review was again specific to the collection of salivary cortisol data in children and may therefore not be representative of all biological data collection. Where large-scale cohort studies do exist, such as the Adolescent Brain Cognitive Development (ABCD) study, specific efforts have been employed to increase retention in this age group, especially where long-term follow-up occurs [29]. There is also an important gap in the distribution of biological research studies in adolescents between low- and middle-income countries (LMIC) and high-income countries (HIC). With the exception of projects

For peer review only

such as the Identifying Depression Early in Adolescence Risk Stratified Cohort (IDEA-RiSCo) recruiting adolescents in Brazil [30], most studies in this area are conducted in HIC, despite the fact that 90% of adolescents worldwide live in LMIC [31,19]. The lack of representatives for cohorts of ethnic minorities and vulnerable populations is particularly concerning given the widening inequalities in children's health and mental health, whereby children from deprived areas are facing the worst of the crisis [32].

Due to these clear barriers to biological research in adolescence and mental health, and the importance of conducting research in this area, it is evident that advancements are needed in the field. While facilitators could be very important to this advancement, there is little dedicated research into possible facilitators for conducting research in this population. Facilitators to adolescents seeking help in a care setting are more widely reported, and mental health literacy, particularly having more information surrounding mental health, was reported as a facilitator [33]. In a research setting, Warne et al., (2022) note that engaging with stakeholders serves as a facilitator, similarly as it helps to increase understanding of the research and topic area, as well as improving protocols and recruitment [21].

Given these limitations, and lack of focus addressing the facilitators for improvement, the CELEBRATE project, co-led in equal partnership by academics and young people, aims to produce a Framework of Guiding Principles as a methodological tool that will operationalise young people's and stakeholders' preferred approach for successfully engaging in biological research in mental health. This work will produce a methodological tool to facilitate biological research in large and representative mental health studies in young people, integrating multiple biological measures, to enhance our ability to draw mechanistic insights into adolescent mental health. Being active decision-makers regarding their own role when participating in research, it is essential for young people to be engaged throughout such work to co-create the knowledge. A plain language summary, accessible for young people, can be found in the Supplementary material.

It is therefore essential to identify the needs of both the population and the research, and the barriers to the research to address and overcome these in future research. There is also value in exploring possible existing, or perceived, facilitators to further aid the development of this important research subject. A preliminary search of PROSPERO, Cochrane Database of Systematic Reviews, Open Science Framework, JBI Evidence Synthesis, and Medline Ovid revealed no current or ongoing scoping or systematic reviews on this topic.

The aim of this scoping review is to gather the current evidence of how to best collect biological data from adolescent participants in mental health research, identifying barriers and facilitators affecting research in this field.

REVIEW QUESTION:

What are the needs of the population and of the research, the barriers, and facilitators, in the collection of biological data in mental health research from adolescent populations?

ELIGIBILITY CRITERIA:

- Inclusion criteria:
    - Studies focussing on people aged 11 to 18 years
    - Research focusing on mental health or psychopathology outcomes and experiences

For peer review only

- Research that includes biological data collection methods, including but not limited to, neuroimaging or biological sample collection.

- Exclusion criteria:
  - Papers not written in the English language
  - Papers without a description of data collection

Context

Studies conducted globally will be included in the review. Samples will not be selected based on cultural, ethnicity or sex/gender-based factors.

Types of sources

This scoping review will consider all study designs, including both qualitative and quantitative methodologies. Reviews will be included, and opinion papers will be included if they specifically discuss the collection of biological data in this group.

METHODS:

This review will use the methodological framework of Levac et al., (2010) [1], supplemented by subsequent recommendations from the Joanna Briggs Institute Scoping Review Methodology [2].

Search strategy

The search strategy will aim to locate published studies only. Search terms have been identified using the PEO (patient, exposure, outcome) Framework which identified the population as young people/adolescents/teenagers/ages 11-18; the exposure as participation in mental health research with biological measurements such as MRI and blood tests; and the outcome as attitudes and beliefs about participation, barriers to recruitment, retention, or involvement (see Appendix I for full search term).

Systematic searches will be undertaken through PubMed, Medline, Scopus, Cochrane, ERIC, EMBASE, ProQuest, EBSCO Global Health electronic databases, and manual exploration of relevant publications, as well as identifying studies through relevant citations. The search strategy, including all identified keywords and index terms, will undergo minor revision as per the requirements of the databases. Two reviewers will independently conduct the search to ensure that the search is accurate and identifies all relevant publications across databases.

Studies published in the English language will be included and there will not be a time restriction on when they were published.

Study/Source of Evidence Selection

Following the search, all identified citations will be collated and uploaded into Mendeley with duplicates removed. A pilot test will be conducted, with a random subset of 10 papers from the search selected for the independent reviewers to assess against the inclusion criteria, to be checked for consistency. Following the pilot, the Titles and Abstracts will then be screened by two independent reviewers, with a third reviewer to resolve discrepancies. Reviewers may also screen relevant 'discussion' sections, due to the nature of the research question, as the broader interpretation of results in context may be reported here. Potentially relevant sources will be reviewed in full and assessed against the inclusion and exclusion criteria by the two reviewers.

For peer review only

Reasons for exclusion will be recorded and reported in a Preferred Reporting Items for Systematic Reviews and Meta-analyses extension for scoping review (PRISMA-ScR) flow chart [34] in the scoping review report. Disagreements between the two reviewers will be resolved by the third reviewer.

Data extraction

Data will be extracted from papers included in the scoping review by two independent reviewers. A third reviewer will decide on any discrepancies in selection. A data extraction form will be developed by the reviewers and will include details of the design, including socio-demographics of samples, recruitment and retention, biological measures, and outcomes. Key issues to investigate include engagement strategies with young people, partnerships with other key stakeholders, cultural appropriateness of research processes, community engagement, feedback strategies, and benefits to schools. Information will be charted on whether strategies were evaluated, appeared to work or not, and why. The process of charting the data will be iterative, with reviewers constantly revising the form to ensure that all relevant data is extracted. Modifications will be detailed in the scoping review report. Specific questions may emerge during the review, such as those focussing on culturally appropriate strategies for recruitment, study advocacy in relation to cultural and linguistic diversity, and more.

A draft extraction form is provided in Appendix II.

Patient and Public Involvement Statement

Patient and Public Involvement (PPI) is at the centre of the CELEBRATE project, of which this scoping review is a part. Our project proposal was developed in consultation with young people and the project as a whole is being co-led by young people. Co-leadership is facilitated through regular meetings with the Youth Expert Working Group (YEWG) as well as the provision of opportunities to provide direct input into project-related activities, such as the approval of search terms specifically relating to this scoping review protocol. Other key stakeholders are also involved in the co-design and governance which includes one of our co-authors for this protocol paper.

Members of our YEWG supported the development of this proposal through a series of meetings in which we discussed search terms and anticipated findings, and supporting the writing of our Plain Language Summary, found in the Supplementary Material.

DATA ANALYSIS AND PRESENTATION:

Data will be summarised in tabular form with a narrative synthesis. We will perform subgroup analysis and/or reporting as data allows.

ETHICS AND DISSEMINATION:

Ethical approval is not required to conduct this scoping review. The scoping review will be conducted with input from Patient and Public Involvement (PPI), specifically including young people involved in our Youth Expert Working Group who will provide feedback on the review findings and interpretation.

Dissemination will include publication in peer-reviewed journals, academic presentations and on the project website www.celebrateproject.co.uk. The results of the scoping review will inform other

For peer review only

aspects of the wider project, including topic guides for focus groups with stakeholders on the topic area.

Word count (excluding Title, Author information, Abstract, Acknowledgements, References and Appendices): 2689

Acknowledgements

Throughout this project, we have worked with members of the CELEBRATE Youth Expert Working Group (YEWG). This group is formed of young people aged 11-16 with vested interest in youth mental health research. Through online meetings, we engaged YEWG members in conversations and activities that provided us an insight into their views on how we can best conduct this research. Their input was particularly valuable in refining the terms we used to conduct this scoping review.

This research is supported by the National Institute for Health Research (NIHR) Mental Health Biomedical Research Centre (BRC) at South London and Maudsley NHS Foundation Trust. This research is supported by the NIHR Oxford Health Biomedical Research Centre. The views expressed are those of the author(s) and not necessarily those of the NIHR or the Department of Health and Social Care.

Funding

This work is being supported by the Medical Research Centre [grant number MR/X003078/1].

Conflicts of interest

None to declare.

Author contributions:

CW has contributed significantly to the drafting and editing of the manuscript. RU, PD, VM and CN are leading this work, conceiving the idea, and developing the study methods. SH has advised on methodology. RP, TW, ZS, MP, AW and RU have contributed to the development of the search strategy, which was discussed with all authors, including the CELEBRATE YEWG. The CELEBRATE YEWG, RP, PD and NC supported the development of the plain language summary. NC has led and facilitated YEWG meetings that have discussed this work, which the CELEBRATE YEWG, CW, RP, ZS, NG, DR, AW, PD and RU have contributed to. All authors have contributed meaningfully to the drafting and editing of the manuscript.

References:

1. Levac D, Colquhoun H, O'Brien KK. Scoping studies: advancing the methodology. Implementation science. 2010 Dec;5:1-9. https://doi.org/10.1186/1748-5908-5-69
2. Peters MD, Godfrey C, McInerney P, Munn Z, Tricco AC, Khalil H. Scoping reviews. Joanna Briggs Institute reviewer's manual. 2017: 1-24.
3. Bonnie RJ, Stroud C. Investing in the health and well-being of young adults. Washington, DC: National Academies Press. 2017.
4. Kieling C, Baker-Henningham H, Belfer M, Conti G, Ertem I, Omigbodun O, Rohde LA, Srinath S, Ulkuer N, Rahman A. Child and adolescent mental health worldwide: evidence for action. The Lancet. 2011 Oct 22;378(9801):1515-25. https://doi.org/10.1016/S0140-6736(11)60827-1

For peer review only

5. Sadler K, Vizard T, Ford T, Marchesell F, Pearce N, Mandalia D, Davis J, Brodie E, Forbes N, Goodman A, Goodman R. Mental health of children and young people in England, 2017. NHS digital, 2018: 1-35.
6. Cybulski L, Ashcroft DM, Carr MJ, Garg S, Chew-Graham CA, Kapur N, Webb RT. Temporal trends in annual incidence rates for psychiatric disorders and self-harm among children and adolescents in the UK, 2003–2018. BMC psychiatry. 2021 Dec;21:1-2. https://doi.org/10.1186/s12888-021-03235-w
7. Racine N, McArthur BA, Cooke JE, Eirich R, Zhu J, Madigan S. Global prevalence of depressive and anxiety symptoms in children and adolescents during COVID-19: a meta-analysis. JAMA pediatrics. 2021 Nov 1;175(11):1142-50. doi:10.1001/jamapediatrics.2021.2482
8. Benton TD, Boyd RC, Njoroge WF. Addressing the global crisis of child and adolescent mental health. JAMA pediatrics. 2021 Nov 1;175(11):1108-10. doi:10.1001/jamapediatrics.2021.2479
9. World Health Organisation. Adolescent mental health. 2021. https://www.who.int/news-room/fact-sheets/detail/adolescent-mental-health
10. Kessler RC, Amminger GP, Aguilar-Gaxiola S, Alonso J, Lee S, Üstün TB. Age of onset of mental disorders: a review of recent literature. Current opinion in psychiatry. 2007 Jul 1;20(4):359-64. *DOI:* 10.1097/YCO.0b013e32816ebc8c
11. Chapman DP, Dube SR, Anda RF. Adverse childhood events as risk factors for negative mental health outcomes. Psychiatric annals. 2007 May 1;37(5):359.
12. Mills R, Scott J, Alati R, O'Callaghan M, Najman JM, Strathearn L. Child maltreatment and adolescent mental health problems in a large birth cohort. Child abuse & neglect. 2013 May 1;37(5):292-302. https://doi.org/10.1016/j.chiabu.2012.11.008
13. Kumpulainen K, Räsänen E. Children involved in bullying at elementary school age: their psychiatric symptoms and deviance in adolescence: an epidemiological sample. Child abuse & neglect. 2000 Dec 1;24(12):1567-77. https://doi.org/10.1016/S0145-2134(00)00210-6
14. Turner MG, Exum ML, Brame R, Holt TJ. Bullying victimization and adolescent mental health: General and typological effects across sex. Journal of Criminal Justice. 2013 Jan 1;41(1):53-9. https://doi.org/10.1016/j.jcrimjus.2012.12.005
15. Moore GF, Cox R, Evans RE, Hallingberg B, Hawkins J, Littlecott HJ, Long SJ, Murphy S. School, peer and family relationships and adolescent substance use, subjective wellbeing and mental health symptoms in Wales: A cross sectional study. Child indicators research. 2018 Dec;11:1951-65. https://doi.org/10.1007/s12187-017-9524-1
16. Cheng Y, Li X, Lou C, Sonenstein FL, Kalamar A, Jejeebhoy S, Delany-Moretlwe S, Brahmbhatt H, Olumide AO, Ojengbede O. The association between social support and mental health among vulnerable adolescents in five cities: findings from the study of the well-being of adolescents in vulnerable environments. Journal of Adolescent Health. 2014 Dec 1;55(6):S31-8. https://doi.org/10.1016/j.jadohealth.2014.08.020
17. Reiss F. Socioeconomic inequalities and mental health problems in children and adolescents: a systematic review. Social science & medicine. 2013 Aug 1;90:24-31. https://doi.org/10.1016/j.socscimed.2013.04.026
18. Medical Research Council. Maximising the value of UK population cohorts MRC Strategic Review of the Largest UK Population Cohort Studies. 2014. https://www.ukri.org/wp-content/uploads/2021/12/MRC-23122021-UK-Population-cohorts.pdf
19. Zajkowska Z, Walsh A, Zonca V, Gullett N, Pedersen GA, Kieling C, Swartz JR, Karmacharya R, Fisher HL, Kohrt BA, Mondelli V. A systematic review of the association between biological markers and environmental stress risk factors for adolescent depression. Journal of psychiatric research. 2021 Jun 1;138:163-75. https://doi.org/10.1016/j.jpsychires.2021.04.003

20. Vogel A, Comtesse H, Rosner R. Challenges in recruiting and retaining adolescents with abuse-related posttraumatic stress disorder: lessons learned from a randomized controlled trial. Child and adolescent psychiatry and mental health. 2020 Dec;14:1-3. https://doi.org/10.1186/s13034-020-00320-y
21. Warne N, Rook S, Jones RB, Brown R, Bates L, Hopkins-Jones L, Evans A, Hall J, Langley K, Thapar A, Walters J. Collecting genetic samples and linked mental health data from adolescents in schools: protocol coproduction and a mixed-methods pilot of feasibility and acceptability. BMJ open. 2022 Feb 1;12(2):e049283. doi: 10.1136/bmjopen-2021-049283
22. Gattuso J, Hinds P, Tong X, Srivastava K. Monitoring child and parent refusals to enrol in clinical research protocols. Journal of advanced nursing. 2006 Feb;53(3):319-26. https://doi.org/10.1111/j.1365-2648.2006.03724.x
23. Youn S, Mamsa S, Allott K, Berger M, Polari A, Rice S, Schmaal L, Wood S, Lavoie S. Acceptability and feasibility of a multidomain harmonized data collection protocol in youth mental health. Early intervention in psychiatry. 2023 May;17(5):512-8. https://doi.org/10.1111/eip.13346
24. Mascarell Maričić L, Walter H, Rosenthal A, Ripke S, Quinlan EB, Banaschewski T, Barker GJ, Bokde AL, Bromberg U, Büchel C, Desrivières S. The IMAGEN study: a decade of imaging genetics in adolescents. Molecular Psychiatry. 2020 Nov;25(11):2648-71. https://doi.org/10.1038/s41380-020-0822-5
25. Dimolareva M, Gee NR, Pfeffer K, Maréchal L, Pennington K, Meints K. Measuring cortisol in the classroom with school-aged children—a systematic review and recommendations. International Journal of Environmental Research and Public Health. 2018 May;15(5):1025. https://doi.org/10.3390/ijerph15051025
26. Gabbay V, Klein RG, Alonso CM, Babb JS, Nishawala M, De Jesus G, Hirsch GS, Hottinger-Blanc PM, Gonzalez CJ. Immune system dysregulation in adolescent major depressive disorder. Journal of affective disorders. 2009 May 1;115(1-2):177-82. https://doi.org/10.1016/j.jad.2008.07.022
27. Condon EM. Psychosocial influences on acceptability and feasibility of salivary cortisol collection from community samples of children. Research in Nursing & Health. 2016 Dec;39(6):449-62. https://doi.org/10.1002/nur.21744
28. Najman JM, Clavarino A, McGee TR, Bor W, Williams GM, Hayatbakhsh MR. Timing and chronicity of family poverty and development of unhealthy behaviors in children: a longitudinal study. Journal of Adolescent Health. 2010 Jun 1;46(6):538-44. https://doi.org/10.1016/j.jadohealth.2009.12.001
29. Ewing SW, Chang L, Cottler LB, Tapert SF, Dowling GJ, Brown SA. Approaching retention within the ABCD study. Developmental cognitive neuroscience. 2018 Aug 1;32:130-7. https://doi.org/10.1016/j.dcn.2017.11.004
30. Kieling C, Buchweitz C, Caye A, Manfro P, Pereira R, Viduani A, Anes M, Battel L, Benetti S, Fisher HL, Karmacharya R. The identifying depression early in adolescence risk stratified cohort (IDEA-RiSCo): rationale, methods, and baseline characteristics. Frontiers in psychiatry. 2021 Jun 21;12:697144. https://doi.org/10.3389/fpsyt.2021.697144
31. Battel L, Cunegatto F, Viduani A, Fisher HL, Kohrt BA, Mondelli V, Swartz JR, Kieling C. Mind the brain gap: the worldwide distribution of neuroimaging research on adolescent depression. Neuroimage. 2021 May 1;231:117865. https://doi.org/10.1016/j.neuroimage.2021.117865
32. Mahase, E. Child health is in crisis as government ignores widening inequalities, paediatricians warn. BMJ. 2023. 381:765. doi: https://doi.org/10.1136/bmj.p765
33. Aguirre Velasco A, Cruz IS, Billings J, Jimenez M, Rowe S. What are the barriers, facilitators and interventions targeting help-seeking behaviours for common mental

health problems in adolescents? A systematic review. BMC psychiatry. 2020 Dec;20(1):1-22. https://doi.org/10.1186/s12888-020-02659-0

34. Tricco AC, Lillie E, Zarin W, O'Brien KK, Colquhoun H, Levac D, Moher D, Peters MD, Horsley T, Weeks L, Hempel S. PRISMA extension for scoping reviews (PRISMA-ScR): checklist and explanation. Annals of internal medicine. 2018 Oct 2;169(7):467-73. https://doi.org/10.7326/M18-0850

For peer review only

APPENDICES:

Appendix I: Search strategy for PubMed database

1. ("MRI" OR "fMRI" OR "EEG" OR "PET" OR "neuroimaging" OR "biological research" OR "biological sample*" OR "biologic*" OR "blood sample* " OR "urine sample*")

AND

2. ("mental health" OR "mental" OR "depression" OR "anxiety" OR "self harm" OR "self-harm" OR "suicide" OR "psychiatric" OR "psychopathology" OR "mood disorder" OR "autism" OR "neurodivergence")

AND

3. ("youth*" OR "adolescent" OR "adolescence" OR "teen*" OR "teenager*" OR "high school student*" OR "AYA")

AND

4. ("retention" OR "recruitment" OR "recruit" OR "recruited" OR "enroll" OR "enrol" OR "enrollment" OR "enrolment" OR "enrolling" OR "enrolled" OR "participation")

Appendix II: Data extraction form

CELEBRATE – WP1 Scoping review data extraction template (version 1)

Date form completed (dd/mm/yyyy)	
Reviewer name	
Citation	

Authors	
Year of publication	
Country of origin	
Indication (eg/ depression or anxiety)	
Aims	
Methodology/study design	
Sample size	
Sample age	
Sample socio-demographics	
Biological measures (list)	
Biological outcomes (list)	
Recruitment details	
Retention details (no. of withdrawals)	
Young people engagement strategies described	
Stakeholder engagement described	
Community engagement described	
Feedback strategies described	

Notes:	

For peer review only

For peer review only

SUPPLEMENTARY MATERIAL:
Plain language summary

This plain language summary has been co-written with young people from our Youth Expert Working Group

Adolescence happens between the ages of 10 and 19 when moving from childhood to adulthood. Adolescence is a time when a person goes through many changes both physically and mentally and is the time when a person may be more vulnerable to having mental health problems, such as depression and/or anxiety. There are lots of things that can increase the chances of mental health problems happening, like genetics, stressful things happening during childhood, or coming from a disadvantaged background. But we still don't know much about how biology plays a role in all of this.

Scientists have done some biological research into mental health during adolescent years, but it's quite limited for different reasons such as the groups who fund research being concerned that young people will not want to take part. Because of this, we don't fully understand how biology mixes with other things like stress, environment, and social factors in adolescence, and to do this we need more research. Therefore, it's important for us to understand what is stopping us from doing this kind of research, and how we can make it easier for young people, like you, to get involved.

We are doing a ‘scoping review’, which is looking at all the studies published previously, to help us to find out more about this issue. A scoping review looks at how much research has been done on the topic and whether there are any gaps in our understanding. This paper explains how we will conduct this review (the so-called “protocol”).

We will be searching different databases to find studies that focus on mental health, and that have looked at people between the ages of 11 and 18. We are especially interested in studies that have collected biological information like blood samples or brain scans.

We will look at all the studies that are relevant and see how they relate to the question we are trying to answer. We will be looking at things like how hard it is to get young people to participate in biological studies, how scientists work with communities, and why some groups of people might be more or less likely to take part in this type of research than others. We will also look for gaps in our knowledge, where none of the previous studies have answered certain questions on this topic.

All of the research we find will be drawn together and summarised to help us understand what we know so far about the difficulties of doing research with young people looking at biology and mental health.

For peer review only

BMJ Open

Exploring the research needs, barriers, and facilitators to the collection of biological data in adolescence for mental health research: A Scoping Review Protocol paper

Journal:	*BMJ Open*
Manuscript ID	bmjopen-2023-081360.R1
Article Type:	Protocol
Date Submitted by the Author:	19-Apr-2024
Complete List of Authors:	Worrell, Courtney ; King's College London, Department of Psychological Medicine, Institute of Psychiatry, Psychology, and Neuroscience<br>Pollard, Rebecca; King's College London, Department of Psychological Medicine, Institute of Psychiatry, Psychology, and Neuroscience<br>Weetman, Tyler; University of Birmingham, Institute for Mental Health<br>Sadiq, Zara; University of Birmingham, Institute for Mental Health<br>Pieptan, Maria; King's College London, Department of Psychological Medicine, Institute of Psychiatry, Psychology, and Neuroscience<br>Brooks, Gillian; King's College London, King's Business School<br>Broome, Matthew ; University of Birmingham, Institute for Mental Health ; Birmingham Women's and Children's NHS Foundation Trust<br>Campbell, Niyah; University of Birmingham, Institute for Mental Health<br>Gardner, Nzinga; NIHR, Public Review Member<br>Harding, Seeromanie; King's College London, Department of Population Health Sciences, Faculty of Life Sciences and Medicine<br>Lavis, Anna; University of Birmingham, Institute of Applied Health Research<br>McEachan, Rosemary; Bradford Institute for Health Research<br>Mondelli, Valeria; King's College London, Department of Psychological Medicine, Institute of Psychiatry, Psychology, and Neuroscience<br>Morgan, Craig; King's College London, Health Service and Population Research Department, Institute of Psychiatry, Psychology, and Neuroscience; King's College London, ESRC Centre for Society and Mental Health, Institute of Psychiatry, Psychology, and Neuroscience<br>Nosarti, Chiara; King's College London, Department of Child and Adolescent Psychiatry, Institute of Psychiatry, Psychology and Neuroscience; King's College London, Centre for the Developing Brain, Department of Perinatal Imaging and Health, School of Biomedical Engineering and Imaging Sciences<br>Porat, Talya; King's College London, Department of Population Health Sciences, Faculty of Life Sciences and Medicine; Imperial College London, Dyson School of Design Engineering, Faculty of Engineering<br>Ryan, David; Bradford Institute for Health Research<br>Schmid, Lea; King's College London, Department of Psychological Medicine, Institute of Psychiatry, Psychology, and Neuroscience<br>Shire, Katy; Bradford Institute for Health Research<br>Woods, Anthony; King's College London, Department of Psychological Medicine, Institute of Psychiatry, Psychology, and Neuroscience<br>Pariante, Carmine; King's College London, Department of Psychological

	Medicine, Institute of Psychiatry, Psychology, and Neuroscience<br>CELEBRATE Youth Expert Working Group , CELEBRATE Youth Expert Working Group ; CELEBRATE project<br>Dazzan, Paola; King's College London, Department of Psychological Medicine, Institute of Psychiatry, Psychology, and Neuroscience<br>Upthegrove, Rachel; University of Birmingham, Institute for Mental Health; Birmingham Women's and Children's NHS Foundation Trust

**Primary Subject Heading**:	Mental health
Secondary Subject Heading:	Mental health
Keywords:	MENTAL HEALTH, Adolescents < Adolescent, Adolescent

SCHOLARONE™
Manuscripts

Exploring the research needs, barriers, and facilitators to the collection of biological data in adolescence for mental health research: A Scoping Review **Protocol paper**

Courtney Worrell[1], Rebecca Pollard[1], Tyler Weetman[2], Zara Sadiq[2], Maria Pieptan[1], Gillian Brooks[3], Matthew R Broome[2,4], Niyah Campbell[2], Nzinga Gardner[5], Seeromanie Harding[6], Anna Lavis[7], Rosemary R C McEachan[8], Valeria Mondelli[1], Craig Morgan[9,10], Chiara Nosarti[11,12], Talya Porat[6,13], David Ryan[8], Lea Schmid[1], Katy Shire[8], Anthony Woods[1], Carmine M. Pariante[1], CELEBRATE Youth Expert Working Group, Paola Dazzan[1], Rachel Upthegrove[2,4]

Affiliations:

1. Department of Psychological Medicine, Institute of Psychiatry, Psychology, and Neuroscience, King's College London, London, UK
2. Institute for Mental Health, University of Birmingham, Birmingham, UK
3. King's Business School, King's College London, London, UK
4. Birmingham Women's and Children's NHS Foundation Trust, Birmingham, UK
5. National Institute for Health and Care Research (NIHR) Public Review Member, UK
6. Department of Population Health Sciences, Faculty of Life Sciences and Medicine, King's College London, London, UK
7. Institute of Applied Health Research, University of Birmingham, Birmingham, UK
8. Bradford Institute of Health Research, Bradford, UK
9. Health Service and Population Research Department, Institute of Psychiatry, Psychology, and Neuroscience, King's College London, London, UK
10. ESRC Centre for Society and Mental Health, Institute of Psychiatry, Psychology, and Neuroscience, King's College London, London, UK
11. Department of Child and Adolescent Psychiatry, Institute of Psychiatry, Psychology and Neuroscience, King's College London, London, UK
12. Centre for the Developing Brain, Department of Perinatal Imaging and Health, School of Biomedical Engineering and Imaging Sciences, King's College London, London, UK
13. Dyson School of Design Engineering, Faculty of Engineering, Imperial College London, London, UK

Author emails:

courtney.worrell@kcl.ac.uk
rebecca.pollard@kcl.ac.uk
tyler.weetman@nhs.net
zara.sadiq4@nhs.net
maria.pieptan@kcl.ac.uk
gillian.brooks@kcl.ac.uk
m.r.broome@bham.ac.uk
n.campbell@bham.ac.uk
nzinga.g@gmail.com
seeromanie.harding@kcl.ac.uk
a.c.lavis@bham.ac.uk
Rosie.McEachan@bthft.nhs.uk
valeria.mondelli@kcl.ac.uk
craig.morgan@kcl.ac.uk

chiara.nosarti@kcl.ac.uk
t.porat@imperial.ac.uk
David.Ryan@bthft.nhs.uk
lea.schmid@kcl.ac.uk
Katy.Shire@bthft.nhs.uk
tony.woods@kcl.ac.uk
carmine.pariante@kcl.ac.uk
paola.dazzan@kcl.ac.uk
r.upthegrove@bham.ac.uk

Corresponding author details: Courtney Worrell, Desk G.33.74, The Maurice Wohl Clinical Neuroscience Institute, Cutcombe road, King's College London, London, SE5 9RT. Email: courtney.worrell@kcl.ac.uk, Tel: 0207 848 5440.

ABSTRACT

Introduction: While research into adolescent mental health has developed a considerable understanding of environmental and psychosocial risk factors, equivalent biological evidence is lacking and is not representative of economic, social, and ethnic diversity in the adolescent population. It is important to understand the possible barriers and facilitators to conduct this research. This will then allow us to improve our understanding of how biology interacts with environmental and psychosocial risk factors during adolescence. The objective of this scoping review is to identify and understand the needs, barriers, and facilitators related to the collection of biological data in adolescent mental health research. **Methods and analysis:** Reviewers will conduct a systematic search of PubMed, Medline, Scopus, Cochrane, ERIC, EMBASE, ProQuest, EBSCO Global Health electronic databases, relevant publications, and reference lists to identify studies published in the English language at any time. This scoping review will identify published studies exploring mental health/psychopathology outcomes, with biological measures, in participants between the ages of 11-18, and examine the reported methodology used for data collection. Data will be summarised in tabular form with narrative synthesis and will use the methodology of Levac et al., (2010), supplemented by subsequent recommendations from the Joanna Briggs Institute Scoping Review Methodology. **Ethics and dissemination:** Ethical approval is not required for this scoping review. The scoping review will be conducted with input from Patient and Public Involvement (PPI), specifically including young people involved in our study 'CELEBRATE' ('Co-producing a framework of guiding principles for Engaging representative and diverse cohorts of young people in Biological ReseArch in menTal hEalth' - www.celebrateproject.co.uk) Youth Expert Working Group. Dissemination will include publication in peer-reviewed journals, academic presentations, and on the project website.

STRENGTHS AND LIMITATIONS OF THIS STUDY

- This review is being conducted according to methodological frameworks and guidance such as that reported by Levac et al., (2010) and the Joanna Briggs Institute Scoping Review Methodology.
- The systematic search will include grey literature which reduces publication bias.
- The search will include 7 different databases (as well as a manual search of the references) to maximise identification of relevant studies.
- The iterative approach to charting data during extraction ensures that all relevant information will be captured.

For peer review only

- The search is limited to work published in the English language which may exclude some relevant publications.

KEYWORDS: Adolescence; Barriers; Biological measures; Facilitators; Mental health.

INTRODUCTION

Adolescence is a distinct period of significant change. Between the ages of 10-19, adolescence sees a string of changes biologically, socially, and psychologically. In this important timeframe, the development that adolescents undergo has an undeniable impact on longer-term trajectories of health and well-being [1]. Due to these substantial changes, this period provides a window of vulnerability, whereby adolescents may be at increased risk of developing poor mental health.

Mental health problems are a leading cause of health-related disability in children and adolescents, with reports that at least 10% globally experience a mental disorder [2]. The UK Office for National Statistics (2017) suggest that 1 in 8 young people between the ages of 5-19 have experienced at least one form of psychopathology in the UK [3]. In a large UK-based cohort, Cybulski et al., (2021) have reported an increased incidence in the number of young people seeking support for mental health disorders between 2003-2018 [4], suggesting an upward trend, one that has seemingly accelerated in the context of COVID-19. Indeed, following the COVID-19 pandemic, concerns about declining adolescent mental health are now being highlighted in the literature, with a meta-analysis indicating a two-fold increase in symptoms of anxiety and depression [5]. It is important to note, however, that approximately half of the studies included in this meta-analysis represent samples from China, and others representing North America and Asia, leading to arguments that they may not represent the global picture, and those from low- and middle-income countries [6]. According to the World Health Organisation (2021), depression, anxiety, and behavioural disorders, such as Attention Deficit Hyperactivity Disorder (ADHD), are the most common forms of mental disorders experienced by adolescents [7]. Regarding mental disorders in adulthood, the available evidence suggests 75% have onset during adolescence [8] showing clear evidence that adolescence is a critical window, and vulnerability during this time can have a substantial impact on longer-term health, specifically mental health. Poor mental health during this period can additionally have an impact on education, future employment, and other longer-term consequences.

Researchers have explored the possible risk factors for mental health disorders in adolescents, focussing on genetic, environmental, and psychosocial risk factors. Some of these factors include exposure to childhood trauma [9,10], bullying [11,12], and difficult family relationships [13], all of which have been shown to play a role in increasing the risk of developing a mental health problem during adolescence. Other factors include environmental and socioeconomic inequalities [14]. A systematic review including data from 23 countries demonstrated that children and adolescents with socioeconomic disadvantage (based on factors such as household income and poverty), were two to three times more likely to develop mental health difficulties, with a somewhat cumulative effect [15].

Existing large adolescent cohorts with mental health data tend to lack biological measures. Existing cohorts typically explore only saliva or small-volume blood samples for genotyping and basic biomarkers [16]. So, whilst this literature begins to demonstrate why adolescents may develop mental health disorders, there is a considerable gap in knowledge from a biological perspective. Specifically, in our understanding of how biology interacts with environmental and psychosocial risk factors to impact mental health during adolescence [17]. Due to the high incidence of mental health

For peer review only

disorders occurring during adolescence, it has been argued that it is important to explore biological markers at this early stage for understanding the development of mental health disorders [18]. Biological evidence, such as fluid-based samples (blood, saliva, urine) and brain imaging can therefore be an invaluable addition to research in adolescent mental health as we aim to advance knowledge of biological mechanisms, treatment and stratification.

It is known that adolescents may be resistant to engaging with mental health services, particularly for trauma-related disorders, which has led to assumptions regarding their willingness to become involved in research [19]. Where studies are beginning to explore richer biological measures, recruitment to such studies may encounter several barriers. A school-based pilot study, conducted by Warne et al., (2022) collecting salivary cortisol samples found that only 11.3% of parents provided consent for their child to participate, despite the coproduction of the protocol with key stakeholders including parents and children [20], demonstrating some of the possible barriers which can affect this research. When research in child and adolescent populations includes biological data collection, lower recruitment rates may be due to the burden and acceptability of the research; in a study exploring child and adolescent participation in research, Gattuso et al., (2006) found high rates of refusal to participate, which often related to the use of data collection methods, such as blood sampling, which were perceived to be invasive and burdensome [21]. In a feasibility study, young people were asked to report their subjective experience after completing mock clinical interviews, self-report forms, neuroimaging, and blood samples [22]. Most responses indicated that the young people felt safe in all the assessments, and the assessment viewed most favourably was the mock magnetic resonance imaging (MRI) scan, which the authors noted as an important finding given the assumption that in research settings MRI is perceived as burdensome and distressing [22]. The mock interviews and self-report measures, on the other hand, were often reported as stressful and too long [22]. The topic of research being considered 'too sensitive' was another highly reported reason for refusal which may imply further difficulties when the topic of research surrounds mental ill health [21]. Some studies also demonstrate issues with levels of attrition in such research. Mascarell Maričić et al., (2020) discuss this issue in the context of the IMAGEN cohort, a longitudinal study of 2000 14-year-olds recruited and followed up to the age of 22. While discussing how attrition can have an impact on longitudinal multimodal statistical modelling, the authors note that they were able to still have complete data for 1300 of the cohort by age 22 which provided this particular study an opportunity to still explore the developmental trajectories in focus [23]. However, a review by Dimolareva et al., (2018) argued that it is generally difficult to understand more about the nature of recruitment challenges, non-compliance, and attrition in research with adolescents as many relevant papers fail to report such information [24].

Even in the face of good recruitment targets, a lack of representativeness still hinders the generalisability of findings to adolescent populations and potentially those most at risk. For example, Warne et al., (2022) acknowledged a lack of diversity in the sample recruited in the salivary cortisol study [20]. Predominantly, research looking at biological mechanisms of mental disorders has focused on adults, and there is a sparsity of biological research on mental disorders, such as depression, in adolescence [25]. Looking at the acceptability of biological data collection in children, Condon (2016) highlighted that those who drop out of research or do not participate efficiently, are possibly those who are most important to investigate, tending to be those from under-represented groups [26], and other research highlights that higher levels of attrition are seen in those with lower socioeconomic status or mental health difficulties [27]. However, it should be noted that this review was again specific to the collection of salivary cortisol data in children and may therefore not be representative of all biological data collection. Where large-scale cohort studies do exist, such as the Adolescent Brain Cognitive Development (ABCD) study, specific efforts have been employed to

For peer review only

increase retention in this age group, especially where long-term follow-up occurs [28]. There is also an important gap in the distribution of biological research studies in adolescents between low- and middle-income countries (LMIC) and high-income countries (HIC). With the exception of projects such as the Identifying Depression Early in Adolescence Risk Stratified Cohort (IDEA-RiSCo) recruiting adolescents in Brazil [29], most studies in this area are conducted in HIC, despite the fact that 90% of adolescents worldwide live in LMIC [30,17]. The lack of representatives for cohorts of ethnic minorities and vulnerable populations is particularly concerning given the widening inequalities in children's health and mental health, whereby children from deprived areas are facing the worst of the crisis [31].

Due to these clear barriers to biological research in adolescence and mental health, and the importance of conducting research in this area, it is evident that advancements are needed in the field. While facilitators could be very important to this advancement, there is little dedicated research into possible facilitators for conducting research in this population. Facilitators to adolescents seeking help in a care setting are more widely reported, and mental health literacy, particularly having more information surrounding mental health, was reported as a facilitator [32]. In a research setting, Warne et al., (2022) note that engaging with stakeholders serves as a facilitator, similarly as it helps to increase understanding of the research and topic area, as well as improving protocols and recruitment [20].

Given these limitations, and lack of focus addressing the facilitators for improvement, the CELEBRATE project, co-led in equal partnership by academics and young people, aims to produce a Framework of Guiding Principles as a methodological tool that will operationalise young people's and stakeholders' preferred approach for successfully engaging in biological research in mental health. This work will produce a methodological tool to facilitate biological research in large and representative mental health studies in young people, integrating multiple biological measures, to enhance our ability to draw mechanistic insights into adolescent mental health. Being active decision-makers regarding their own role when participating in research, it is essential for young people to be engaged throughout such work to co-create the knowledge. A plain language summary, accessible for young people, can be found in the Supplementary material.

It is therefore essential to identify the needs of both the population and the research, and the barriers to the research to address and overcome these in future research. There is also value in exploring possible existing, or perceived, facilitators to further aid the development of this important research subject. A preliminary search of PROSPERO, Cochrane Database of Systematic Reviews, Open Science Framework, JBI Evidence Synthesis, and Medline Ovid revealed no current or ongoing scoping or systematic reviews on this topic.

The aim of this scoping review is to gather the current evidence of how to best collect biological data (such as brain imaging and fluid-based samples) from adolescent participants in mental health research, identifying barriers and facilitators affecting research in this field.

REVIEW QUESTION:

What are the needs of the population and of the research, the barriers, and facilitators, in the collection of biological data in mental health research from adolescent populations?

METHODS:

For peer review only

This review will use the methodological framework of Levac et al., (2010) [33], supplemented by subsequent recommendations from the Joanna Briggs Institute (JBI) Scoping Review Methodology [34]. The JBI guidance is a further refined set of guidance building on that of Levac et al., specifically focussing on rigorous synthesis. The JBI Manual for Evidence Synthesis [35] has been referred to in the preparation of this work. The design of this work began in early 2023, with the initial search performed in February 2023. The work is scheduled to finish at the end of 2024.

Search strategy

The search strategy will aim to locate published studies only. Search terms have been identified using the PEO (patient, exposure, outcome) Framework which identified the population as young people/adolescents/teenagers/ages 11-18; the exposure as participation in mental health research with biological measurements such as MRI and blood tests; and the outcome as attitudes and beliefs about participation, barriers to recruitment, retention, or involvement (see Appendix I for full search term).

Systematic searches will be undertaken through PubMed, Medline, Scopus, Cochrane, ERIC, EMBASE, ProQuest, EBSCO Global Health electronic databases, and manual exploration of relevant publications, as well as identifying studies through relevant citations. The addition of grey literature aims to reduce the potential influence of publication bias and improve the comprehensiveness of the review, allowing us to identify as much of the relevant literature as possible. The use of grey literature in reviews is important [36]. The search strategy, including all identified keywords and index terms, will undergo minor revision as per the requirements of the databases. Two reviewers will independently conduct the search to ensure that the search is accurate and identifies all relevant publications across databases.

Studies published in the English language will be included and there will not be a time restriction on when they were published.

ELIGIBILITY CRITERIA:

- Inclusion criteria:
  - Studies focussing on people aged 11 to 18 years (studies which recruit participants within this age range will be included even if younger or older participants are also included, for example, child and adolescent samples, and adolescent and young adult samples). Samples which had children up to 11 are not included and samples which recruited adults who are 18 and above are not included).
  - Research focusing on mental health or psychopathology outcomes and experiences
  - Research that includes biological data collection methods, including but not limited to, neuroimaging or biological sample collection.

- Exclusion criteria:
  - Papers not written in the English language
  - Papers without a description of data collection

Context

Studies conducted globally will be included in the review. Samples will not be selected based on cultural, ethnicity or sex/gender-based factors.

For peer review only

Types of sources

This scoping review will consider all study designs, including both qualitative and quantitative methodologies. Reviews will be included, and opinion papers will be included if they specifically discuss the collection of biological data in this group.

Study/Source of Evidence Selection

Following the search, all identified citations will be collated and uploaded into Zotero with duplicates removed. A pilot test will be conducted, with a random subset of 10 papers from the search selected for the independent reviewers to assess against the inclusion criteria, to be checked for consistency. Following the pilot, the Titles and Abstracts will then be screened by two independent reviewers, with a third reviewer to resolve discrepancies. Reviewers may also screen relevant 'discussion' sections, due to the nature of the research question, as the broader interpretation of results in context may be reported here. Potentially relevant sources will be reviewed in full and assessed against the inclusion and exclusion criteria by the two reviewers. Reasons for exclusion will be recorded and reported in a Preferred Reporting Items for Systematic Reviews and Meta-analyses extension for scoping review (PRISMA-ScR) flow chart [37] in the scoping review report. Disagreements between the two reviewers will be resolved by the third reviewer.

Data extraction

Data will be extracted from papers included in the scoping review by reviewers, with an independent reviewer checking the consistency of extraction. A data extraction form will be developed by the reviewers and will include details of the design, including socio-demographics of samples, recruitment and retention, biological measures, and outcomes. Key issues to investigate include engagement strategies with young people, partnerships with other key stakeholders, cultural appropriateness of research processes, community engagement, feedback strategies, and benefits to schools. Information will be charted on whether strategies were evaluated, appeared to work or not, and why. The process of charting the data will be iterative, with reviewers constantly revising the form to ensure that all relevant data is extracted. Modifications will be detailed in the scoping review report. Specific questions may emerge during the review, such as those focussing on culturally appropriate strategies for recruitment, study advocacy in relation to cultural and linguistic diversity, and more.

A draft extraction form is provided in Appendix II.

Patient and Public Involvement Statement

Patient and Public Involvement (PPI) is at the centre of the CELEBRATE project, of which this scoping review is a part. Our project proposal was developed in consultation with young people and the project as a whole is being co-led by young people. Co-leadership is facilitated through regular meetings with the Youth Expert Working Group (YEWG) as well as the provision of opportunities to provide direct input into project-related activities, such as the approval of search terms specifically relating to this scoping review protocol. Other key stakeholders are also involved in the co-design and governance which includes one of our co-authors for this protocol paper.

Members of our YEWG supported the development of this proposal through a series of meetings in which we discussed search terms and anticipated findings, and supporting the writing of our Plain Language Summary, found in the Supplementary Material.

For peer review only

DATA ANALYSIS AND PRESENTATION:

Data will be summarised in tabular form with a narrative synthesis. We will perform subgroup analysis and/or reporting as data allows.

ETHICS AND DISSEMINATION:

Ethical approval is not required to conduct this scoping review. The scoping review will be conducted with input from Patient and Public Involvement (PPI), specifically including young people involved in our Youth Expert Working Group who will provide feedback on the review findings and interpretation.

Dissemination will include publication in peer-reviewed journals, academic presentations and on the project website www.celebrateproject.co.uk. The results of the scoping review will inform other aspects of the wider project, including topic guides for focus groups with stakeholders on the topic area.

Word count (excluding Title, Author information, Abstract, Acknowledgements, References and Appendices): 2930

Acknowledgements

Throughout this project, we have worked with members of the CELEBRATE Youth Expert Working Group (YEWG). This group is formed of young people aged 11-16 with vested interest in youth mental health research. Through online meetings, we engaged YEWG members in conversations and activities that provided us an insight into their views on how we can best conduct this research. Their input was particularly valuable in refining the terms we used to conduct this scoping review.

This research is supported by the National Institute for Health Research (NIHR) Mental Health Biomedical Research Centre (BRC) at South London and Maudsley NHS Foundation Trust. This research is supported by the NIHR Oxford Health Biomedical Research Centre. The views expressed are those of the author(s) and not necessarily those of the NIHR or the Department of Health and Social Care.

Funding

This work is being supported by the Medical Research Centre [grant number MR/X003078/1].

Conflicts of interest

None to declare.

Author contributions:

CW has contributed significantly to the drafting and editing of the manuscript. RU, PD, VM and CN are leading this work, conceiving the idea, and developing the study methods. SH has advised on methodology. RP, TW, ZS, MP, AW, CW and RU have contributed to the development of the search strategy, which was discussed with all authors, including GB, MB, NC, NG, SH, AL, RRCM, VM, CM, CN, TP, DR, LS, KS, CMP, CELEBRATE YEWG, and PD. The CELEBRATE YEWG, RP, PD and NC supported the development of the plain language summary. NC has led and facilitated YEWG meetings that have discussed this work, which the CELEBRATE YEWG, CW, RP, ZS, NG, DR, AW, PD and RU have

For peer review only

contributed to. All authors, CW, RP, TW, ZS, MP, GB, MB, NC, NG, SH, AL, RRCM, VM, CM, CN, TP, DR, LS, KS, AW, CMP, CELEBRATE YEWG, PD and RU, have contributed meaningfully to the drafting and editing of the manuscript.

References:

1. Bonnie RJ, Stroud C. Investing in the health and well-being of young adults. Washington, DC: National Academies Press. 2017.
2. Kieling C, Baker-Henningham H, Belfer M, Conti G, Ertem I, Omigbodun O, Rohde LA, Srinath S, Ulkuer N, Rahman A. Child and adolescent mental health worldwide: evidence for action. The Lancet. 2011 Oct 22;378(9801):1515-25. https://doi.org/10.1016/S0140-6736(11)60827-1
3. Sadler K, Vizard T, Ford T, Marchesell F, Pearce N, Mandalia D, Davis J, Brodie E, Forbes N, Goodman A, Goodman R. Mental health of children and young people in England, 2017. NHS digital, 2018: 1-35.
4. Cybulski L, Ashcroft DM, Carr MJ, Garg S, Chew-Graham CA, Kapur N, Webb RT. Temporal trends in annual incidence rates for psychiatric disorders and self-harm among children and adolescents in the UK, 2003–2018. BMC psychiatry. 2021 Dec;21:1-2. https://doi.org/10.1186/s12888-021-03235-w
5. Racine N, McArthur BA, Cooke JE, Eirich R, Zhu J, Madigan S. Global prevalence of depressive and anxiety symptoms in children and adolescents during COVID-19: a meta-analysis. JAMA pediatrics. 2021 Nov 1;175(11):1142-50. doi:10.1001/jamapediatrics.2021.2482
6. Benton TD, Boyd RC, Njoroge WF. Addressing the global crisis of child and adolescent mental health. JAMA pediatrics. 2021 Nov 1;175(11):1108-10. doi:10.1001/jamapediatrics.2021.2479
7. World Health Organisation. Adolescent mental health. 2021. https://www.who.int/news-room/fact-sheets/detail/adolescent-mental-health
8. Kessler RC, Amminger GP, Aguilar-Gaxiola S, Alonso J, Lee S, Üstün TB. Age of onset of mental disorders: a review of recent literature. Current opinion in psychiatry. 2007 Jul 1;20(4):359-64. *DOI:* 10.1097/YCO.0b013e32816ebc8c
9. Chapman DP, Dube SR, Anda RF. Adverse childhood events as risk factors for negative mental health outcomes. Psychiatric annals. 2007 May 1;37(5):359.
10. Mills R, Scott J, Alati R, O'Callaghan M, Najman JM, Strathearn L. Child maltreatment and adolescent mental health problems in a large birth cohort. Child abuse & neglect. 2013 May 1;37(5):292-302. https://doi.org/10.1016/j.chiabu.2012.11.008
11. Kumpulainen K, Räsänen E. Children involved in bullying at elementary school age: their psychiatric symptoms and deviance in adolescence: an epidemiological sample. Child abuse & neglect. 2000 Dec 1;24(12):1567-77. https://doi.org/10.1016/S0145-2134(00)00210-6
12. Turner MG, Exum ML, Brame R, Holt TJ. Bullying victimization and adolescent mental health: General and typological effects across sex. Journal of Criminal Justice. 2013 Jan 1;41(1):53-9. https://doi.org/10.1016/j.jcrimjus.2012.12.005
13. Moore GF, Cox R, Evans RE, Hallingberg B, Hawkins J, Littlecott HJ, Long SJ, Murphy S. School, peer and family relationships and adolescent substance use, subjective wellbeing and mental health symptoms in Wales: A cross sectional study. Child indicators research. 2018 Dec;11:1951-65. https://doi.org/10.1007/s12187-017-9524-1
14. Cheng Y, Li X, Lou C, Sonenstein FL, Kalamar A, Jejeebhoy S, Delany-Moretlwe S, Brahmbhatt H, Olumide AO, Ojengbede O. The association between social support and mental health among vulnerable adolescents in five cities: findings from the study of the

well-being of adolescents in vulnerable environments. Journal of Adolescent Health. 2014 Dec 1;55(6):S31-8. https://doi.org/10.1016/j.jadohealth.2014.08.020

15. Reiss F. Socioeconomic inequalities and mental health problems in children and adolescents: a systematic review. Social science & medicine. 2013 Aug 1;90:24-31. https://doi.org/10.1016/j.socscimed.2013.04.026
16. Medical Research Council. Maximising the value of UK population cohorts MRC Strategic Review of the Largest UK Population Cohort Studies. 2014. https://www.ukri.org/wp-content/uploads/2021/12/MRC-23122021-UK-Population-cohorts.pdf
17. Zajkowska Z, Walsh A, Zonca V, Gullett N, Pedersen GA, Kieling C, Swartz JR, Karmacharya R, Fisher HL, Kohrt BA, Mondelli V. A systematic review of the association between biological markers and environmental stress risk factors for adolescent depression. Journal of psychiatric research. 2021 Jun 1;138:163-75. https://doi.org/10.1016/j.jpsychires.2021.04.003
18. Quinlan EB, Banaschewski T, Barker GJ, Bokde AL, Bromberg U, Büchel C, Desrivieres S, Flor H, Frouin V, Garavan H, Heinz A. Identifying biological markers for improved precision medicine in psychiatry. Molecular psychiatry. 2020 Feb;25(2):243-53. https://doi.org/10.1038/s41380-019-0555-5
19. Vogel A, Comtesse H, Rosner R. Challenges in recruiting and retaining adolescents with abuse-related posttraumatic stress disorder: lessons learned from a randomized controlled trial. Child and adolescent psychiatry and mental health. 2020 Dec;14:1-3. https://doi.org/10.1186/s13034-020-00320-y
20. Warne N, Rook S, Jones RB, Brown R, Bates L, Hopkins-Jones L, Evans A, Hall J, Langley K, Thapar A, Walters J. Collecting genetic samples and linked mental health data from adolescents in schools: protocol coproduction and a mixed-methods pilot of feasibility and acceptability. BMJ open. 2022 Feb 1;12(2):e049283. doi: 10.1136/bmjopen-2021-049283
21. Gattuso J, Hinds P, Tong X, Srivastava K. Monitoring child and parent refusals to enrol in clinical research protocols. Journal of advanced nursing. 2006 Feb;53(3):319-26. https://doi.org/10.1111/j.1365-2648.2006.03724.x
22. Youn S, Mamsa S, Allott K, Berger M, Polari A, Rice S, Schmaal L, Wood S, Lavoie S. Acceptability and feasibility of a multidomain harmonized data collection protocol in youth mental health. Early intervention in psychiatry. 2023 May;17(5):512-8. https://doi.org/10.1111/eip.13346
23. Mascarell Maričić L, Walter H, Rosenthal A, Ripke S, Quinlan EB, Banaschewski T, Barker GJ, Bokde AL, Bromberg U, Büchel C, Desrivières S. The IMAGEN study: a decade of imaging genetics in adolescents. Molecular Psychiatry. 2020 Nov;25(11):2648-71. https://doi.org/10.1038/s41380-020-0822-5
24. Dimolareva M, Gee NR, Pfeffer K, Maréchal L, Pennington K, Meints K. Measuring cortisol in the classroom with school-aged children—a systematic review and recommendations. International Journal of Environmental Research and Public Health. 2018 May;15(5):1025. https://doi.org/10.3390/ijerph15051025
25. Gabbay V, Klein RG, Alonso CM, Babb JS, Nishawala M, De Jesus G, Hirsch GS, Hottinger-Blanc PM, Gonzalez CJ. Immune system dysregulation in adolescent major depressive disorder. Journal of affective disorders. 2009 May 1;115(1-2):177-82. https://doi.org/10.1016/j.jad.2008.07.022
26. Condon EM. Psychosocial influences on acceptability and feasibility of salivary cortisol collection from community samples of children. Research in Nursing & Health. 2016 Dec;39(6):449-62. https://doi.org/10.1002/nur.21744
27. Najman JM, Clavarino A, McGee TR, Bor W, Williams GM, Hayatbakhsh MR. Timing and chronicity of family poverty and development of unhealthy behaviors in children: a

longitudinal study. Journal of Adolescent Health. 2010 Jun 1;46(6):538-44. https://doi.org/10.1016/j.jadohealth.2009.12.001
28. Ewing SW, Chang L, Cottler LB, Tapert SF, Dowling GJ, Brown SA. Approaching retention within the ABCD study. Developmental cognitive neuroscience. 2018 Aug 1;32:130-7. https://doi.org/10.1016/j.dcn.2017.11.004
29. Kieling C, Buchweitz C, Caye A, Manfro P, Pereira R, Viduani A, Anes M, Battel L, Benetti S, Fisher HL, Karmacharya R. The identifying depression early in adolescence risk stratified cohort (IDEA-RiSCo): rationale, methods, and baseline characteristics. Frontiers in psychiatry. 2021 Jun 21;12:697144. https://doi.org/10.3389/fpsyt.2021.697144
30. Battel L, Cunegatto F, Viduani A, Fisher HL, Kohrt BA, Mondelli V, Swartz JR, Kieling C. Mind the brain gap: the worldwide distribution of neuroimaging research on adolescent depression. Neuroimage. 2021 May 1;231:117865. https://doi.org/10.1016/j.neuroimage.2021.117865
31. Mahase, E. Child health is in crisis as government ignores widening inequalities, paediatricians warn. BMJ. 2023. 381:765. doi: https://doi.org/10.1136/bmj.p765
32. Aguirre Velasco A, Cruz IS, Billings J, Jimenez M, Rowe S. What are the barriers, facilitators and interventions targeting help-seeking behaviours for common mental health problems in adolescents? A systematic review. BMC psychiatry. 2020 Dec;20(1):1-22. https://doi.org/10.1186/s12888-020-02659-0
33. Levac D, Colquhoun H, O'Brien KK. Scoping studies: advancing the methodology. Implementation science. 2010 Dec;5:1-9. https://doi.org/10.1186/1748-5908-5-69
34. Peters MD, Godfrey C, McInerney P, Munn Z, Tricco AC, Khalil H. Scoping reviews. Joanna Briggs Institute reviewer's manual. 2017: 1-24.
35. Aromataris E, Munn Z. JBI Manual for Evidence Synthesis. JBI. 2020. https://synthesismanual.jbi.global. https://doi.org/10.46658/JBIMES-20-01
36. Paez A. Gray literature: An important resource in systematic reviews. Journal of Evidence-Based Medicine. 2017 Aug;10(3):233-240. doi: 10.1111/jebm.12266.
37. Tricco AC, Lillie E, Zarin W, O'Brien KK, Colquhoun H, Levac D, Moher D, Peters MD, Horsley T, Weeks L, Hempel S. PRISMA extension for scoping reviews (PRISMA-ScR): checklist and explanation. Annals of internal medicine. 2018 Oct 2;169(7):467-73. https://doi.org/10.7326/M18-0850

For peer review only

APPENDICES:

Appendix I: Search strategy for PubMed database

1. ("MRI" OR "fMRI" OR "EEG" OR "PET" OR "neuroimaging" OR "biological research" OR "biological sample*" OR "biologic*" OR "blood sample* " OR "urine sample*")

AND

2. ("mental health" OR "mental" OR "depression" OR "anxiety" OR "self harm" OR "self-harm" OR "suicide" OR "psychiatric" OR "psychopathology" OR "mood disorder" OR "autism" OR "neurodivergence")

AND

3. ("youth*" OR "adolescent" OR "adolescence" OR "teen*" OR "teenager*" OR "high school student*" OR "AYA")

AND

4. ("retention" OR "recruitment" OR "recruit" OR "recruited" OR "enroll" OR "enrol" OR "enrollment" OR "enrolment" OR "enrolling" OR "enrolled" OR "participation")

Appendix II: Data extraction form

CELEBRATE – WP1 Scoping review data extraction template (version 1)

Date form completed (dd/mm/yyyy)	
Reviewer name	
Citation	

Authors	
Year of publication	
Country of origin	
Indication (eg/ depression or anxiety)	
Aims	
Methodology/study design	
Sample size	
Sample age	
Sample socio-demographics	
Biological measures (list)	
Biological outcomes (list)	
Recruitment details	
Retention details (no. of withdrawals)	
Young people engagement strategies described	
Stakeholder engagement described	
Community engagement described	
Feedback strategies described	

Notes:	

For peer review only

For peer review only

SUPPLEMENTARY MATERIAL:
Plain language summary

This plain language summary has been co-written with young people from our Youth Expert Working Group

Adolescence happens between the ages of 10 and 19 when moving from childhood to adulthood. Adolescence is a time when a person goes through many changes both physically and mentally and is the time when a person may be more vulnerable to having mental health problems, such as depression and/or anxiety. There are lots of things that can increase the chances of mental health problems happening, like genetics, stressful things happening during childhood, or coming from a disadvantaged background. But we still don't know much about how biology plays a role in all of this.

Scientists have done some biological research into mental health during adolescent years, but it's quite limited for different reasons such as the groups who fund research being concerned that young people will not want to take part. Because of this, we don't fully understand how biology mixes with other things like stress, environment, and social factors in adolescence, and to do this we need more research. Therefore, it's important for us to understand what is stopping us from doing this kind of research, and how we can make it easier for young people, like you, to get involved.

We are doing a ‘scoping review’, which is looking at all the studies published previously, to help us to find out more about this issue. A scoping review looks at how much research has been done on the topic and whether there are any gaps in our understanding. This paper explains how we will conduct this review (the so-called “protocol”).

We will be searching different databases to find studies that focus on mental health, and that have looked at people between the ages of 11 and 18. We are especially interested in studies that have collected biological information like blood samples or brain scans.

We will look at all the studies that are relevant and see how they relate to the question we are trying to answer. We will be looking at things like how hard it is to get young people to participate in biological studies, how scientists work with communities, and why some groups of people might be more or less likely to take part in this type of research than others. We will also look for gaps in our knowledge, where none of the previous studies have answered certain questions on this topic.

All of the research we find will be drawn together and summarised to help us understand what we know so far about the difficulties of doing research with young people looking at biology and mental health.

For peer review only
